# Real-World Outcomes of First-Line Cetuximab and Platinum-Based Chemotherapy in Recurrent and/or Metastatic Head and Neck Squamous Cell Carcinoma: A Multicenter Observational Study and Literature Review

**DOI:** 10.3390/curroncol33040183

**Published:** 2026-03-26

**Authors:** Zoran Rakušić, Vesna Bišof, Sanja Vušković, Zdenka Kotromanović, Suzana Erić, Jelena Viculin, Ljubica Vazdar, Marin Prpić, Davor Kust, Damir Vučinić

**Affiliations:** 1 Department of Oncology, University Hospital Center Zagreb, 10000 Zagreb, Croatia; zoran.rakusic@medikol.hr (Z.R.); sanja.vuskovic@kbc-zagreb.hr (S.V.); 2 School of Medicine, University of Zagreb, 10000 Zagreb, Croatia; ljubica.vazdar@kbcsm.hr (L.V.); davor.kust@kbcsm.hr (D.K.); 3 Medikol Polyclinic, 10000 Zagreb, Croatia; 4 Department of Oncology, University Hospital Center Osijek, 31000 Osijek, Croatia; zdenka.kotromanovic@kbco.hr (Z.K.); eric.suzana@kbo.hr (S.E.); 5 Faculty of Medicine, Josip Juraj Strossmayer University of Osijek, 31000 Osijek, Croatia; 6 Faculty of Dental Medicine and Health, University of Osijek, 31000 Osijek, Croatia; 7 Department of Oncology and Radiotherapy, University Hospital Center Split, 21000 Split, Croatia; jelena.viculin@kbsplit.hr; 8 Division of Medical Oncology, University Hospital for Tumors, Sestre Milosrdnice University Hospital Center, 10000 Zagreb, Croatia; 9 Department of Oncology and Nuclear Medicine, Sestre Milosrdnice University Hospital Center, 10000 Zagreb, Croatia; marin.prpic@kbcsm.hr; 10 School of Dental Medicine, University of Zagreb, 10000 Zagreb, Croatia; 11 Tumor Clinic, Clinical Hospital Center Rijeka, 51000 Rijeka, Croatia; damir.vucinic@radiochirurgia.hr; 12 School of Medicine, University of Rijeka, 51000 Rijeka, Croatia; 13 Special Hospital Radiochirurgia Zagreb, 10431 Sveta Nedjelja, Croatia

**Keywords:** head and neck cancer, first-line, cetuximab, real-world evidence, recurrence, metastasis

## Abstract

Background: The EXTREME regimen (cetuximab with cisplatin/carboplatin and 5-fluorouracil) has long been a standard first-line treatment for recurrent and/or metastatic head and neck squamous cell carcinoma (R/M HNSCC), particularly in patients ineligible for immunotherapy. However, real-world evidence remains limited, especially in regions with delayed access to novel therapies. Methods: We conducted a retrospective, multicenter study of 217 patients with R/M HNSCC treated with cetuximab-based chemotherapy at six Croatian oncology centers between 2016 and 2022, prior to reimbursement of pembrolizumab. The primary endpoint was overall survival (OS); secondary endpoints included progression-free survival (PFS), response rates, and safety. Results: The majority (91%) received the EXTREME regimen. Median OS was 14 months (95% CI, 12–17), and median PFS was 6.2 months (95% CI, 6.0–7.2). Objective response rate was 21%, and disease control rate was 63%. Cetuximab-induced rash correlated with longer PFS. Grade ≥ 3 toxicity occurred in 18.9% of patients. No treatment-related deaths were observed. Conclusion: In routine clinical practice, cetuximab combined with platinum-based chemotherapy remains an effective and well-tolerated first-line treatment for R/M HNSCC, particularly in patients who are ineligible for immunotherapy or with PD-L1–negative tumors. These findings support its continued use in appropriately selected patients.

## Simple Summary

Recurrent or metastatic head and neck squamous cell carcinoma is a challenging disease that often requires systemic treatment. For many years, the combination of chemotherapy and the targeted agent cetuximab (the EXTREME regimen) was the standard first-line therapy. Although immunotherapy is now widely used, not all patients are eligible for it, and access may be limited in some countries. In this multicenter retrospective study, we analyzed the outcomes of 217 patients in Croatia treated with cetuximab-based chemotherapy before immunotherapy became available. The treatment provided meaningful survival and disease control with acceptable toxicity in routine clinical practice. Patients who developed cetuximab-related skin rash had better progression-free survival. These findings support the continued use of cetuximab-based chemotherapy as a treatment option for selected patients with recurrent or metastatic head and neck cancer.

## 1. Introduction

Although the incidence and mortality rates of head and neck cancers (HNC) vary across regions worldwide, globally, HNC ranks as the seventh most commonly diagnosed cancer, accounting for more than 890,000 new cases and approximately 450,000 deaths annually [1]. Approximately 90% of these cases are squamous cell carcinomas (SCC). At diagnosis, around 10–15% of patients present with distant metastases, and about 50% have locoregionally advanced disease [2]. Among those with locoregional disease, 50–60% will eventually develop recurrent and/or metastatic disease. Only about 20% of patients are eligible for local treatment modalities. Therefore, systemic therapy remains the cornerstone of management.

From 2008 to 2019, the EXTREME regimen, comprising 5-fluorouracil, cisplatin or carboplatin, and cetuximab, was the standard first-line treatment for patients with recurrent and/or metastatic head and neck squamous cell carcinoma (R/M HNSCC) [3]. In European populations, this regimen significantly improved overall survival (OS) (median 10.1 vs. 7.4 months; hazard ratio [HR] 0.80, 95% confidence interval [CI] 0.64–0.99), progression-free survival (PFS) (5.6 vs. 3.3 months; HR 0.54, 95% CI 0.43–0.67), overall response rate (ORR) (36% vs. 20%), and disease control rate (DCR) (81% vs. 60%) compared with chemotherapy alone. These findings were subsequently corroborated 13 years later in the phase III CHANGE-2 trial conducted in a Chinese population [4]. The efficacy of the EXTREME regimen using carboplatin was further supported by the phase II ELAN-FIT study in fit patients aged 70 years or older, demonstrating an ORR of 40%, DCR of 71.8%, median OS of 15.8 months, and median PFS of 6.0 months [5].

In a phase II study, Guigay et al. [6] compared the TPEx regimen (docetaxel, cisplatin, and cetuximab) with EXTREME. Although no significant differences in efficacy were observed (median OS 14.5 vs. 13.4 months; median PFS 6.0 vs. 6.2 months; ORR 57% vs. 59%), the TPEx regimen was associated with significantly lower rates of grade ≥ 3 toxicity (81% vs. 93%).

Furthermore, HPV status has been shown to have prognostic significance [7,8]. Its predictive role in cetuximab therapy, however, remains inconclusive, although some studies suggest higher objective response rates in HPV-negative compared with HPV-positive tumors [9].

Soulieres et al. [10] demonstrated no significant differences in safety or efficacy between the two cetuximab formulations, and that cisplatin and carboplatin are complementary, with the choice depending on the patient’s clinical profile.

Following the results of the KEYNOTE-048 trial, the U.S. Food and Drug Administration (FDA) approved pembrolizumab in 2019 as monotherapy for tumors expressing programmed death-ligand 1 (PD-L1; combined positive score, CPS ≥ 1) or in combination with 5-fluorouracil and cisplatin/carboplatin regardless of PD-L1 status, as the preferred first-line treatment for R/M HNSCC [11,12]. In the same year, the European Commission approved pembrolizumab, either as monotherapy or in combination with chemotherapy, for PD-L1–positive tumors [13].

Nevertheless, the EXTREME regimen remains a recommended option for patients with PD-L1–negative tumors or those ineligible for immunotherapy, as outlined in the European Society for Medical Oncology (ESMO) guidelines [14]. It also continues to be listed as a treatment option in the current National Comprehensive Cancer Network (NCCN) guidelines [15]. Additionally, the EXTREME regimen may still be preferred in cases involving large or rapidly progressing tumors [16].

However, access to newly approved therapies varies considerably between countries [17]. In Croatia, cetuximab was not reimbursed until 2016, and pembrolizumab, either as monotherapy or in combination with chemotherapy, for first-line treatment of PD-L1 CPS ≥ 1 tumors was not reimbursed until November 2023.

The use of cetuximab in combination with chemotherapy as a first-line treatment for R/M HNSCC has been evaluated in numerous real-world studies aiming to assess treatment outcomes in routine clinical practice outside the controlled settings of clinical trials [3,4,5,6,18,19,20,21,22,23,24,25,26,27,28,29,30,31,32,33,34,35,36,37,38,39,40,41,42] (Table S1). Here, we present the results of a retrospective, observational study conducted across six oncology centers in Croatia.

## 2. Materials and Methods

### 2.1. Data Sources

We conducted a retrospective analysis of data from 217 patients with R/M HNSCC who received first-line treatment with cetuximab in combination with cisplatin-based chemotherapy at the six largest oncology centers in Croatia between March 2016 and January 2022. During this period, pembrolizumab had not yet been approved for the treatment of R/M HNSCC in Croatia. Eligible patients were aged 18 years or older, had histologically confirmed squamous cell carcinoma of the oral cavity, oropharynx, larynx, or hypopharynx, and were deemed ineligible for local therapy.

Data were extracted from hospital information systems and included the following variables: sex, age, Eastern Cooperative Oncology Group (ECOG) performance status, primary tumor site, extent of disease, p16 status (for oropharyngeal cancer), prior treatment in the non-metastatic setting, chemotherapy regimen, type of platinum compound administered, number of chemotherapy cycles, number of cetuximab administrations during maintenance, total number of cetuximab administrations during first-line treatment, time to first radiological assessment, duration of therapy, treatment response, date of disease progression, date of last follow-up or death, and treatment-related adverse events.

p16 status, serving as a surrogate marker for HPV in oropharyngeal cancers, was evaluated in archived tumor tissues by immunohistochemistry using the Ventana Roche CINtec^®^ p16 Histology assay, Tucson, AZ, USA (lot no. N14733). Tumors exhibiting p16 overexpression in ≥70% of tumor cells were classified as positive.

Patients treated according to the EXTREME protocol received 5-fluorouracil (1000 mg/m^2^/day for 4 days) and cisplatin (100 mg/m^2^ on day 1) or carboplatin (target area under the curve [AUC] 5 on day 1) every 21 days, for up to six cycles. Cetuximab was administered on days 1, 8, and 15 (400 mg/m^2^ loading dose on day 1 of cycle 1, followed by 250 mg/m^2^ weekly). During the maintenance phase, cetuximab was administered either weekly at 250 mg/m^2^ in two centers or biweekly at 500 mg/m^2^ in four centers.

Patients treated according to the TPEx protocol received docetaxel (75 mg/m^2^) and cisplatin (75 mg/m^2^), both administered intravenously on day 1 of each 21-day cycle, for up to four cycles. Cetuximab was administered on days 1, 8, and 15 as described above. All patients in the TPEx group received prophylactic granulocyte colony-stimulating factor (G-CSF) support during each cycle. During maintenance phase, cetuximab was administered at a dose of 500 mg/m^2^ every two weeks.

Tumor response was assessed using multi-slice computed tomography (MSCT) (Siemens, Erlangen, Germany) of the head and neck, as well as the chest and abdomen. The cut-off date for follow-up was 30 August 2024.

### 2.2. Endpoints

The primary endpoint was OS, defined as the time from initiation of first-line treatment for R/M HNSCC to death from any cause. For patients still alive at the time of analysis, OS was censored at the date of last follow-up. PFS was defined as the time from initiation of treatment to radiographic disease progression or death from any cause, whichever occurred first.

Treatment response was evaluated according to the Response Evaluation Criteria in Solid Tumors (RECIST), version 1.1 [43], and categorized as complete response (CR), partial response (PR), stable disease (SD), or progressive disease (PD). The ORR was defined as the proportion of patients achieving CR or PR, while the DCR was defined as the proportion of patients achieving CR, PR, or SD.

Adverse events were retrospectively extracted from patients’ medical records, including clinical notes, laboratory results, and treatment documentation. They were assessed according to the Common Terminology Criteria for Adverse Events (CTCAE) version 4.03 or 5.0 [44,45], depending on the period of treatment.

### 2.3. Ethical Considerations

All data were anonymized and centrally analyzed. The study was approved by the ethics committees of all participating institutions (Approval numbers: 02/013 AG, R1-398/2025, 2181-147/01-06/LJ.Z.-25-02, 251-29-11/3-24-20, 251-29-11/3-24-06, 2170-29-02/1-25-2) and was conducted in accordance with the principles of the Declaration of Helsinki [46]. Patient consent was waived due to the retrospective design of the study.

### 2.4. Statistical Analysis

OS and PFS were estimated using the Kaplan–Meier method. Differences between groups were compared using the log-rank test. All statistical tests were two-sided, with a *p*-value < 0.05 considered statistically significant. Subgroup analyses were conducted to evaluate the influence of baseline characteristics and treatment-related variables on OS and PFS using a Cox proportional hazards regression model. Hazard ratios (HRs) and corresponding 95% confidence intervals (CIs) were calculated and presented using forest plots. All statistical analyses were performed using the R statistical computing and graphics environment Version 452.

## 3. Results

### 3.1. Patient Characteristics and Treatment Summary

The median follow-up time was 59 months (95% CI, 57–not reached (NR)). The median age of patients was 61 years (range, 30–88), with a predominance of males (84%). Most patients had an ECOG performance status of 0–1 (91.7%) (Table 1). The majority presented with metastatic disease at the time of treatment initiation (82.5%). Prior chemotherapy in the curative setting had been administered in 51.6% of patients. The EXTREME regimen was used as the first-line treatment of advanced disease in 91% of cases. Cisplatin could not be administered in 13.8% of patients, who were instead treated with carboplatin. Among the 84 patients with oropharyngeal cancer p16 status was positive in 8 patients (9.5%), negative in 25 (29.8%), and unknown in 51 (60.7%).

The median number of chemotherapy cycles administered was 6 (interquartile range (IQR), 4–6). Maintenance therapy was initiated in 119 patients (55%), with a median of 8 cetuximab administrations during the maintenance phase (IQR, 6–14). Among these patients, 52 (44%) received weekly dosing, while 67 patients (56%) received biweekly dosing. The median total number of cetuximab administrations (including both chemotherapy cycles and maintenance therapy) was 18 (IQR, 10–26).

The median duration of total therapy was 22 weeks (IQR, 15–34), and the median duration of maintenance therapy was 7 weeks (IQR, 1–20).

### 3.2. Efficacy of Treatment

A total of 201 patients underwent radiological evaluation of disease status, with a median time to first assessment being 12 weeks (IQR, 10–18). ORR and DCR are summarized in Table 2.

**Figure 1:**
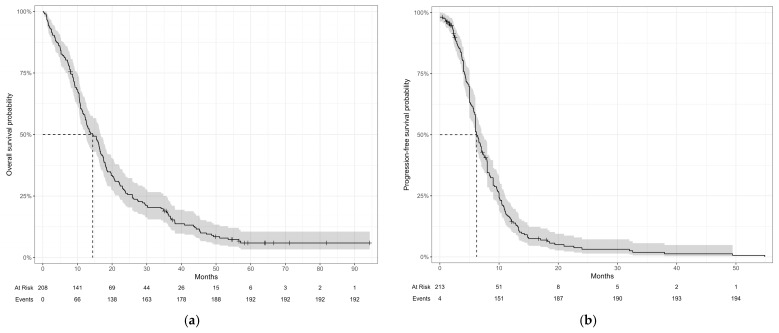
Kaplan–Meier estimates of (**a**) overall survival; (**b**) progression-free survival.

The median OS was 14 months (95% CI, 12–17), and the median PFS was 6.2 months (95% CI, 6.0–7.2) (Figure 1a,b).

**Figure 2:**
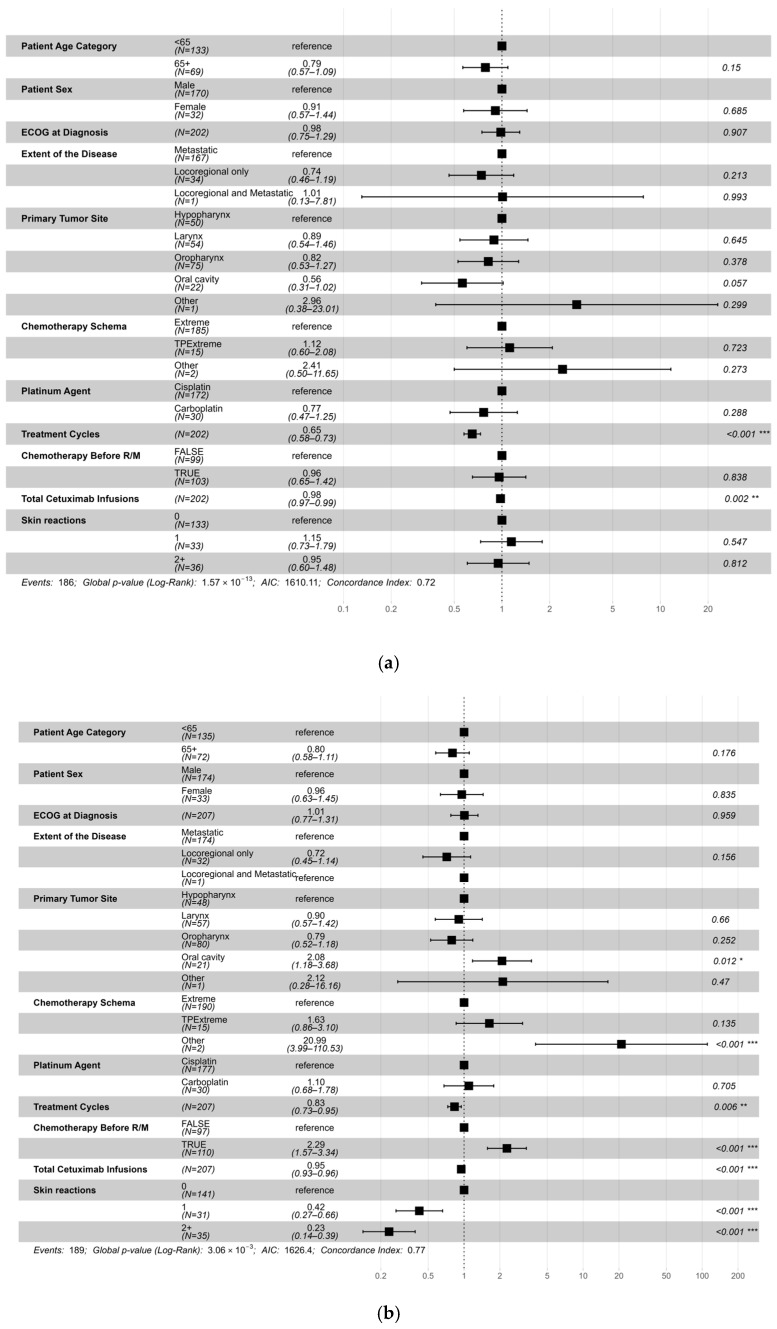
Hazard ratios for (**a**) death; (**b**) disease progression * *p* < 0.05; ** *p* < 0.01; *** *p* < 0.001.

Subgroup analyses revealed that both the number of chemotherapy cycles and the number of cetuximab administrations had a statistically significant impact on OS and PFS. Furthermore, patients who developed a prominent cetuximab-induced skin rash experienced improved PFS, with the magnitude of benefit correlating with the severity of the rash (Figure 2a,b). In contrast, patients who had previously received chemotherapy in the curative setting, as well as those with primary tumors in the oral cavity, demonstrated significantly shorter PFS. In our cohort, survival did not differ between patients receiving weekly and biweekly cetuximab as maintenance therapy (*p* = 0.3).

### 3.3. Safety

Grade 3 and 4 adverse events occurred in 18.9% of patients (Table 3), with grade 4 toxicities observed in 10 patients (4.6%). Cetuximab therapy was permanently discontinued in six patients: five cases (2.3%) due to grade 4 infusion-related reactions, and one case (0.5%) due to grade 4 fatigue. No treatment-related deaths were reported.

## 4. Discussion

In this retrospective, multicenter cohort study, we present real-world outcomes of 217 Croatian patients with R/M HNSCC, the majority of whom received the EXTREME regimen as first-line therapy. Only 6.9% of patients were treated with the TPEx regimen. Consistent with the study by Guigay et al. [6], no significant differences in efficacy were observed between the EXTREME and TPEx regimens. However, it is important to note that the number of patients receiving the TPEx protocol was very limited, and this study was not designed to compare treatment regimens.

The baseline characteristics of our cohort were generally comparable to those reported by Vermorken et al. [3], with several notable differences. Our population included a higher proportion of older patients (≥65 years: 35% vs. 18%), a lower proportion with recurrent-only disease at the time of treatment initiation (16.1% vs. 53%), and a higher proportion treated with cisplatin (85% vs. 67%). In subgroup analyses, neither age, extent of disease nor choice of platinum agent significantly influenced treatment efficacy. Consistent with our observations, both the EXTREME and Soulieres et al. trials demonstrated that substituting cisplatin with carboplatin did not compromise outcomes [3,10]. Similarly, in the DIRECT trial [19], neither age nor extent of disease at baseline adversely affected overall survival.

Regarding primary tumor site, our study demonstrated the highest OS among patients with oral cavity carcinoma, consistent with findings from Vermorken et al. [3], Gao et al. [4], and Pontes et al. [27]. However, in contrast to these studies, PFS for patients with oral cavity tumors was shorter than for other tumor sites in our cohort. This discrepancy may reflect differences in tumor biology, response dynamics, or variability in imaging interpretation.

Maintenance therapy was administered to 55% of patients, similar to the rates reported in the CHANGE-2 and DIRECT trials, and higher than in the EXTREME trial (45%) [3,4,19]. Our findings support previous studies indicating that weekly and biweekly cetuximab maintenance regimens offer comparable efficacy and safety [6,19,20,42].

The median OS of 14 months observed in our study exceeded that reported in the EXTREME trial, and falls within the range of outcomes reported in other randomized and real-world studies. Reported median OS with first-line cetuximab-containing chemotherapy for R/M HNSCC ranges from 7.9 to 17.9 months [3,4,5,6,18,19,20,21,22,23,24,25,26,27,28,29,30,31,32,33,34,35,36,37,38,39,40,41,42]. Similarly, our median PFS of 6.2 months aligns with results from most published studies.

Consistent with Pontes et al. [27], we observed that cetuximab-induced skin rash was associated with improved PFS. Skin rash and mucositis were the most frequently reported adverse events.

Grade 3–4 adverse events occurred in 18.9% of patients, comparable to the 25% reported by Falco et al. [35], but notably lower than the rates observed in the EXTREME (82%) and CHANGE-2 (61.3%) trials [3,4]. However, the incidence of infusion reactions was 3.2%, similar to that reported in the aforementioned two randomized phase III studies. It should be acknowledged that, due to the retrospective design, under-reporting of adverse events, particularly low-grade events, may have occurred.

Compared with the patient population treated with the two cetuximab formulations in Soulieres et al. [10], our cohort included a higher proportion of patients with metastatic disease, while age distribution and male predominance were similar. The overall frequency of adverse events of all grades was comparable, with the most common toxicities in our population being rash, mucositis, and nausea, whereas in the study by Soulieres et al. [10], nausea, fatigue, and hypomagnesemia were most frequently observed. The incidence of higher-grade adverse events was also very similar between the two cohorts.

Although ORR and DCR vary considerably across studies, phase III trials have consistently reported an ORR of approximately 36% for the EXTREME regimen [3,11,47,48]. In contrast, our real-world cohort demonstrated a lower ORR of 21% and a DCR of 63%. Notably, time to treatment failure (TTF) was comparable to that reported in the EXTREME trial, whereas the duration of response in our cohort was nearly twice as long (11.0 vs. 5.6 months). This apparent discrepancy may be attributed to several factors. Although the RECIST v1.1 criteria were applied, response assessments were conducted by different radiologists across participating institutions, and baseline and follow-up imaging were not consistently conducted at the same facility. Additionally, imaging protocols were not standardized across centers, although MSCT was the most frequently used modality. Response evaluations were based on local assessments without centralized radiologic review, which may have introduced inter-observer variability.

Moreover, the higher proportion of patients with metastatic disease at treatment initiation, compared with those with recurrent-only disease, may have contributed to the lower ORR observed. Importantly, real-world evidence studies are inherently prone to multiple sources of bias, including outcome misclassification and non-standardized assessment schedules, as highlighted by methodological frameworks such as APPRAISE [49]. These limitations may lead to an underestimation of categorical endpoints such as ORR in observational settings, whereas time-to-event outcomes, including TTF and duration of response, are generally more robust indicators of clinical benefit.

Other limitations of our study include its retrospective design, lack of data on the relative dose intensity of cetuximab, and missing information regarding second-line therapy, including whether it was administered and which agents were used, all of which may have influenced observed treatment outcomes. In Croatia, nivolumab was approved as the only immunotherapy for second-line treatment of R/M HNSCC in July 2022. Therefore, during the period covered by this study, taxane monotherapy was likely the most commonly used second-line regimen.

In addition, PD-L1 status was not routinely assessed, considering the time period covered by this study, during which pembrolizumab was not yet available in Croatia as a first-line treatment option for R/M HNSCC.

The absence of p16 status data for up to 61% of patients with oropharyngeal cancer represents a notable limitation of this study, as it may have influenced OS, PFS, and ORR.

p16 status is well recognized as a prognostic factor for oropharyngeal carcinomas [8]. Some studies have also suggested that p16-negative tumors may exhibit higher ORR [9]. This challenge is not unique to our cohort and is commonly observed in real-world studies. Among the 19 studies summarized in Table S1, p16 status was reported in only five [31,33,34,37,41], and in two of these, more than 60% of patients had unknown p16 status [31,41].

Notably, the KESTREL study showed that patients who received second-line immunotherapy following first-line EXTREME treatment had longer median OS than those who did not receive immunotherapy or did not receive any second-line treatment [50].

These results should be interpreted within the context of the current post-KEYNOTE 048 treatment landscape. Since pembrolizumab, alone or in combination with chemotherapy, established as a standard first-line therapy for PD-L1 positive R/M HNSCC, cetuximab-based regimens are now primarily relevant for patients who are ineligible for immunotherapy or have PD-L1 negative tumors. Our findings offer real-world evidence supporting the continued effectiveness and tolerability of the EXTREME regimen in these patient populations.

## 5. Conclusions

Cetuximab in combination with platinum-based chemotherapy has demonstrated efficacy not only in well-controlled phase III clinical trials but also in numerous real-world studies. While pembrolizumab, either alone or in combination with chemotherapy, is now the preferred first-line treatment for tumors with PD-L1 CPS ≥ 1, cetuximab plus platinum-based chemotherapy remains a recommended first-line option for patients with PD-L1 negative tumors and those ineligible for immunotherapy. As treatment decisions increasingly rely on patient- and tumor-specific factors, a central challenge in managing R/M HNSCC is optimizing therapy sequencing to maximize clinical outcomes.

Our findings reinforce that first-line treatment with cetuximab and platinum-based chemotherapy continues to be a viable and effective option for a subset of patients, providing manageable toxicity and acceptable adverse event profiles in routine clinical practice.

## Figures and Tables

**Table 1:** Patients’ baseline characteristics and treatment summary.

Characteristics	N = 217 (%)
Age category (yr.)	
<65	142 (65.0)
≥65	75 (35.0)
Sex	
Male	183 (84.0)
Female	34 (16.0)
ECOG PS status	
0	68 (31.3)
1	131 (60.4)
2	18 (8.3)
Primary tumor site	
Hypopharynx	51 (23.5)
Larynx	59 (27.2)
Oropharynx	84 (38.7)
Oral cavity	22 (10.1)
Other	1 (0.5)
Extent of disease	
Locoregionally recurrent only	35 (16.1)
Metastatic and locoregional	3 (1.4)
Metastatic only	179 (82.5)
Previous therapy	200 (92)
Previous chemotherapy	
Yes	112 (51.6)
No	102 (47.0)
Unknown	3 (1.4)
Chemotherapy protocol	
EXTREME	198 (91.2)
TPEx	15 (6.9)
Other	4 (1.8)
Platinum compound	
Cisplatin	185 (85.3)
Carboplatin	30 (13.8)
Unknown	2 (0.9)

yr.—year; ECOG PS—Eastern Cooperative Oncology Group performance status; EXTREME—5-fluorouracil, cisplatin or carboplatin and cetuximab; TPEx—docetaxel, cisplatin and cetuximab.

**Table 2:** Treatment efficacy.

Efficacy	N = 201
Best overall response, N (%)	
Complete response	6 (3)
Partial response	36 (18)
Stable disease	85 (42)
Progressive disease	74 (37)
ORR, N (%)	42 (21)
95% CI	16–27
DCR, N (%)	127 (63)
95% CI	56–70
Median DOR, months	11
95% CI	10–16
Median TTF, months	6.0
95% CI	5.6–6.9

ORR—objective response rate; DCR—disease control rate; CI—confidence interval; DOR—duration of response; TTF—time to treatment failure.

**Table 3:** Overview of adverse events.

Event	Grade 1–2, N (%)	Grade 3, N (%)	Grade 4, N (%)
Any	168 (77.4)	31 (14.3)	10 (4.6)
Skin rash	63 (29.0)	7 (3.2)	1 (0.5)
Mucositis	23 (10.6)	7 (3.2)	0
Nausea	22 (10.1)	2 (0.9)	0
Polyneuropathy	12 (5.5)	0	0
Neutropenia	10 (4.6)	5 (2.3)	2 (0.9)
Hypocalcemia	8 (3.7)	0	0
Vomiting	8 (3.7)	0	0
Hypomagnesemia	5 (2.3)	0	0
Infusion reaction	4 (1.8)	2 (0.9)	5 (2.3)
Leucopenia	3 (1.4)	5 (2.3)	0
Thrombocytopenia	3 (1.4)	0	0
Anemia	2 (0.9)	0	1 (0.5)
Febrile neutropenia	2 (0.9)	2 (0.9)	0
Renal failure	2 (0.9)	0	0
Fatigue	1 (0.5)	0	1 (0.5)
Sepsis	0	1 (0.5)	0

## Data Availability

The data presented in this study are available upon request from the corresponding author. The data are not publicly available due to privacy and ethical restrictions.

## Supplementary Materials

The following supporting information can be downloaded at: https://www.mdpi.com/article/10.3390/curroncol33040183/s1, Table S1: Randomized and real-world studies of cetuximab in the first-line treatment of recurrent and/or metastatic squamous cell carcinoma of the head and neck.

## Abbreviations

The following abbreviations are used in this manuscript:

R/M HNSCC	Recurrent/Metastatic head and neck squamous cell carcinoma	
OS	Overall survival	Directory of open access journals
PFS	Progression-free survival	Three letter acronym
ORR	Objective response rate	Linear dichroism
