# Supplementary material for: Real-World Outcomes of First-Line Cetuximab and Platinum-Based Chemotherapy in Recurrent and/or Metastatic Head and Neck Squamous Cell Carcinoma: A Multicenter Observational Study and Literature Review
Source: Curr Oncol. 2026 Mar 26;33(4):183. doi: 10.3390/curroncol33040183 (PMC13115529; doi:10.3390/curroncol33040183)
Supplement: Supplementary file 1 [file curroncol-33-00183-s001.zip › curroncol-4177726-supplementary.pdf]

Supplementary Materials

Table S1 Randomized and real-world studies of cetuximab in the first-line treatment of recurrent and/or metastatic squamous cell carcinoma of the head and neck

Reference	Study design	N	Treatment protocol	Median OS (mo.)	Median PFS (mo.)	ORR (%)	DCR (%)
	**Randomized trials**						
Vermorken et al., 2008 [3]	phase 3	222	EXTREME	10.1	5.6	36.0	81.0
Guo et al., 2021 [4]	phase 3	164	EXTREME	11.1	5.5	50.0	75.6
Guigay et al., 2024 [5]	phase 2	78 (age 70-89)	EXTREME (carbo)	15.8	6.0	40.0	71.8
Yoshino et al., 2013 [18]	phase 2	33	EXTREME	14.1	4.1	36.0	88.0
Guigay et al., 2022 [19]	phase 4	119	EXTREME	9.4	4.5	-	-
Klinghammer et al., 2019 [20]	phase 2	89<br>91	DPFC<br>EXTREME	8.9<br>10.6	6.3<br>6.4	38.2<br>31.9	71.9<br>68.2
Klinghammer et al., 2021 [21]	phase 2	117<br>123	CF+ tomo<br>EXTREME	11.6<br>13.8	6.5<br>6.2	44.4<br>46.3	75.2<br>76.4
Guigay et al., 2021 [6]	phase 2	271<br>270	TPExtreme<br>EXTREME	14.5<br>13.4	6.0<br>6.2	57.0<br>59.0	83.0<br>80.0
Bossi et al., 2017 [22]	phase 2	100<br>91	CetCis<br>CetCisPac	13.0<br>11.0	6.0<br>7.0	41.8<br>51.7	77.6<br>76.4
Tahara et al., 2018 [23]	phase 2	45	PCC	14.7	5.2	40.0	60.0
	**Real world studies**						
Le Tourneau et al., 2023 [24]	prospective	221	EXTREME 54.8%; Carbo + cetuxi 31.2%	10.8	6.5	27.6	65.6
Hecht et al., 2021 [25]	prospective	128	EXTREME 69%, cis/carbo + cetuxi 23%	7.9	5.1	26	54.2
Yazilitas D et al., 2023 [26]	retrospective	60	EXTREME	9.06	7.0	-	-
Pontes F et al., 2021 [27]	retrospective	103	EXTREME	11.7	7.1	42.7	70.0
Bahl et al., 2019 [28]	retrospective	140	PCC 59%,	11.8	8.1	77.1	85.0

			EXTREME 30%				
Tiwari et al., 2017 [29]	prospective	50	EXTREME	9.93	5.3	50.0	92.0
Chen et al., 2022 [30]	retrospective	107	EXTREME	13.0	5.0	33.7	56.7
Sano et al., 2019 [31]	retrospective	100	EXTREME	11.0	5.0	-	-
Zupančić et al., 2021 [32]	retrospective	33	EXTREME	11.5	7.2	57.5	72.7
Depenni et al., 2019 [33]	retrospective	297	PC 45.3%<br>PC + other 49.7%	10.8	4.8	35.5	-
Nakano et al., 2017 [34]	retrospective	49<br>36	Pacli (w) + cetuxi<br>EXTREME	16.8<br>11.8	6.0<br>5.0	45.0<br>51.0	-
Falco et al., 2022 [35]	retrospective	24	TPExtreme	-	6.9	62.5	87.5
Libert et al., 2025 [36]	retrospective	204	TPExtreme	17.9	6.0	-	-
Simon et al., 2025 [37]	retrospective	56	TPExtreme	13.7	5.9	59	-
Aguin et al., 2023 [38]	retrospective	50 – 1st line	Pacli (w) + cetuxi	8.55	8.55	13.4	60.0
Fushimi et al., 2020 [39]	retrospective	59 (1st line 35)	Pacli (w) + cetuxi	11.8	5.7	46.0	73.0
Rubio-Casadevall et al., 2023 [40]	retrospective	531	Pacli (w) + cetuxi	8.9	4.5	37.7	54.6
Wang et al., 2025 [41]	retrospective	67	CPF4 37.3%<br>CPF2 37.3%	8.7<br>16.2	7.8<br>16.2	-	-
Posch et al., 2016 [42]	retrospective	31	Doce + cetuxi	8.3	4.0	12.9	41.9

mo – months; ORR – objective response rate; DCR – disease control rate; w – weekly; PCC- paclitaxel, carboplatin, cetuximab; PC – cisplatin/carboplatin and 5-fluorouracil; CPF4, cetuximab, cisplatin, 5-fluorouracil every four weeks; CPF2,cetuximab, cisplatin, 5-fluorouracil every two weeks; cetuxi – cetuximab; pacli – paclitaxel; doce – docetaxel; cis – cisplatin; carbo – carboplatin; DPFC – docetaxel, cisplatin, 5-fluorouracil, cetuximab; CF – cisplatin, 5-fluorouracil; tomo - tomozotuximab; CetCis – cetuximab, cisplatin; CetCisPac - cetuximab, cisplatin, paclitaxel
